# Transposable elements in the mammalian embryo: pioneers surviving through stealth and service

**DOI:** 10.1186/s13059-016-0965-5

**Published:** 2016-05-09

**Authors:** Patricia Gerdes, Sandra R. Richardson, Dixie L. Mager, Geoffrey J. Faulkner

**Affiliations:** Mater Research Institute, University of Queensland, TRI Building, Woolloongabba, QLD 4102 Australia; Department of Medical Genetics, Terry Fox Laboratory, British Columbia Cancer Agency, University of British Columbia, Vancouver, BC V5Z 1L3 Canada; School of Biomedical Sciences, University of Queensland, Brisbane, QLD 4072 Australia

## Abstract

Transposable elements (TEs) are notable drivers of genetic innovation. Over evolutionary time, TE insertions can supply new promoter, enhancer, and insulator elements to protein-coding genes and establish novel, species-specific gene regulatory networks. Conversely, ongoing TE-driven insertional mutagenesis, nonhomologous recombination, and other potentially deleterious processes can cause sporadic disease by disrupting genome integrity or inducing abrupt gene expression changes. Here, we discuss recent evidence suggesting that TEs may contribute regulatory innovation to mammalian embryonic and pluripotent states as a means to ward off complete repression by their host genome.

## Background

Mammalian embryonic development is governed by a complex set of genetic and epigenetic instructions. This genomic blueprint undergoes evolutionary selection and, as such, the fundamental order of development is well conserved among mammals. At fertilization, sperm and egg unite to form the zygote, which undergoes successive cleavage divisions, yielding two-, four-, and eight-cell embryonic stages [1, 2]. Initially, the zygotic genome is transcriptionally inactive, with maternally inherited factors regulating embryonic metabolism and development. Embryonic genome activation occurs at around the eight-cell stage in humans and the two-cell stage in mice [3] and is accompanied in each species by epigenome-wide remodeling [4]. The zygote and its daughter cells are totipotent; that is, they have the potential to differentiate into all embryonic and extraembryonic cell types. During development, the differentiation potential of embryonic cells becomes progressively more restricted. At the blastocyst stage, the cells of the inner cell mass (ICM) are pluripotent, meaning that while they cannot give rise to extraembryonic tissues, they can generate all cell lineages and are able to self-renew. Hence, early development involves rapid cellular diversification driven by myriad, and largely still undefined, transcriptional and epigenetic programs (Box 1).

**Fig. 1:**
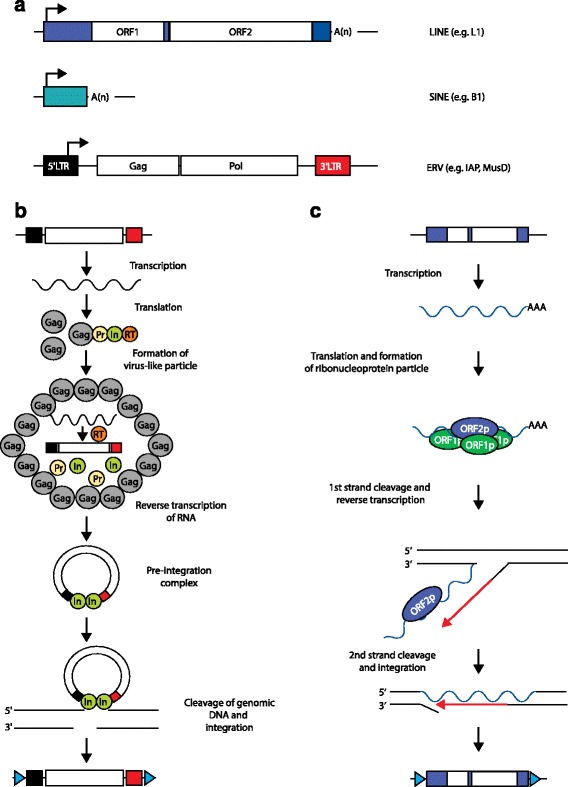
Long terminal repeat (*LTR*) and non-LTR retrotransposition mechanisms. **a** Mammalian retrotransposon structures. A long interspersed element (*LINE*; human L1 shown) typically consists of a 5′ untranslated region (UTR; *blue box*) harboring an internal promoter, two open reading frames (*ORF1*, *ORF2*), a 3′ UTR (*small blue box*), and a poly(A)-tail. A short interspersed element (*SINE*; mouse B1 shown) does not encode proteins and is *trans*-mobilized by LINE proteins. An endogenous retrovirus (*ERV*), such as mouse intracisternal A-type particle (*IAP*) and Mus type-D related retrovirus (*MusD*), lacks an Env protein but encodes functional Gag and Pol proteins flanked by a LTR at the 5′ (*black box*) and 3′ (*red box*) ends. *Arrows* indicate transcription start sites. **b** ERV mobilization starts with mRNA transcription and translation to yield Gag and Gag–Pro–Pol fusion proteins. The fusion proteins consist of a Gag protein (*Gag*), a protease (*Pr*), an integrase (*In*), and a reverse transcriptase (*RT*). Gag proteins build a virus-like particle and encapsulate the fusion proteins, which are processed into separate mature proteins. The ERV mRNA is then reverse transcribed, generating a cDNA. This cDNA and the integrase build a preintegration complex. The integrase then creates a double-strand DNA break, followed by genomic integration of a new ERV copy. Target site duplications (TSDs) are indicated by *blue triangles*. **c** L1 mobilization begins with transcription of an L1 mRNA, which is translated to yield ORF1p and ORF2p. ORF1p, ORF2p, and the L1 mRNA form a ribonucleoprotein particle that re-enters the nucleus. The ORF2p endonuclease cleaves the first genomic DNA strand, while its reverse transcriptase uses a now free 3′ OH group as a primer for reverse transcription of the L1 mRNA. Following second-strand DNA cleavage, a new L1 copy is integrated into the genome and is typically flanked by TSDs

Pluripotent states arising embryonically in vivo, or achieved in vitro by cellular reprogramming, are associated with epigenetic derepression and transcriptional activation of transposable elements (TEs) [4–6]. These mobile genetic elements are found in every eukaryotic genome sequenced to date and account for at least half of mammalian DNA [7–9]. In most mammals, retrotransposons are the predominant TEs. These can be divided into long terminal repeat (LTR) retrotransposons, including endogenous retroviruses (ERVs), and non-LTR retrotransposons such as long interspersed elements (LINEs) and short interspersed elements (SINEs) (Fig. 1a) [10–12]. LINE-1 (L1; Box 2), and ERV families are the only autonomous retrotransposons identified in the human and mouse genomes, though, importantly, human ERVs (HERVs) are all likely now retrotransposition incompetent (Box 3).

**Fig. 2:**
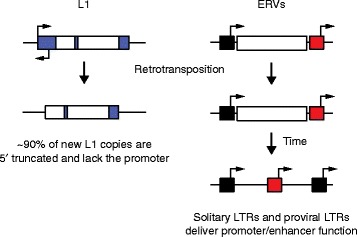
Long interspersed element 1 (*L1*) and endogenous retrovirus (*ERV*) regulatory impact post-integration. Most L1 copies are 5′ truncated (*left*) and lack the sense and antisense L1 promoters located in the 5′ untranslated region (*large blue box*). As a result, these L1 insertions have less capacity to drive chimeric transcription with neighboring genes. ERV insertions (*right*) remain either full-length, with flanking 5′ (*black box*) and 3′ long terminal repeats (*LTRs*; *red box*) that potentially retain promoter function, or, more commonly, recombine between the LTRs to form a solitary LTR, which retains the promoter/enhancer region. *Arrows* indicate putative transcription start sites

All retrotransposons mobilize via a “copy-and-paste” mechanism involving a transcribed RNA intermediate that is reverse transcribed and integrated as a nascent cDNA into genomic DNA. However, there are essential differences in the retrotransposition mechanisms used by LTR and non-LTR retrotransposons (Fig. 1b, c). L1 mRNA transcription relies on an internal 5′ promoter, whereas ERV proviruses utilize a 5′ LTR promoter for transcription initiation (Fig. 1a). Crucially, most new L1 insertions are 5′ truncated and therefore lack the core L1 regulatory sequence. Of 500,000 human L1 copies, only about 7000 retain the canonical 5′ promoter [7, 13]. By contrast, about 90 % of HERVs exist in the genome as solitary LTRs due to recombination of proviral 5′ and 3′ LTRs [11, 14]. Many of these LTRs maintain, or restore through acquired mutations, their natural transcriptional and regulatory signatures, which can perturb the expression of nearby genes [15]. While the regulatory capacity of older LTRs will tend to diminish over time, the approximately 440,000 identifiable LTRs in the human genome [7] still carry enormous potential to regulate genes and gene networks [14–17]. Therefore, compared with L1, ERVs are arguably a much greater source of regulatory innovation (Fig. 2).

**Fig. 3:**
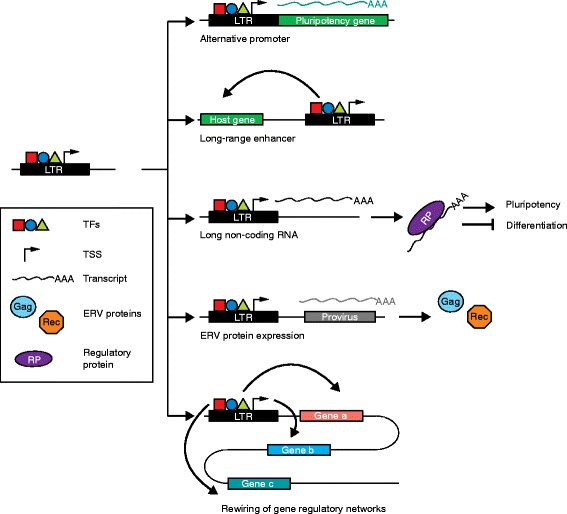
Examples of endogenous retrovirus (*ERV*) contributions to pluripotency. A long terminal repeat (*LTR*) possesses binding sites for pluripotency transcription factors (*TFs*) and can serve as a transcription start site (*TSS*). LTRs bound by pluripotency TFs can thereby impact embryonic stem cell identity by: (1) serving as alternative promoters for pluripotency genes, (2) providing long-range enhancers to specific host genes, (3) generating stem-cell-specific long noncoding RNAs that can bind to proteins regulating the pluripotent state, (4) transcribing proviral DNA elements as precursors to ERV protein expression, and (5) rewiring gene regulatory networks by controlling several pluripotency genes

Recent studies have revealed a complex and somewhat paradoxical interplay between retrotransposons and their host genome in pluripotent cells. On one hand, retrotransposons have long been regarded as fundamentally selfish genetic elements [18] that, to ensure their survival, must evade host genome surveillance and mobilize in cells that provide opportunities for germline transmission. Transcriptional reactivation of retrotransposons in the early mammalian embryo aligns with this evolutionary imperative, despite retrotransposition posing a threat to genome integrity. Indeed, cells employ numerous mechanisms to restrict retrotransposition at this stage [19–23]. On the other hand, transcription from ERV promoters drives the expression of cellular genes as well as ERV-derived sequences and appears to be a fundamental characteristic of the pluripotent state [16, 24–31]. LTRs may be permitted to thrive in this environment due to the materials they provide to the host genome for regulatory network innovation (Fig. 3). Indeed, as well as providing alternative promoters to pluripotency genes [28], ERVs can serve as long-range enhancers [26], produce regulatory noncoding RNAs [27, 30], and may, in some cases, express their own viral proteins [29, 31]. Hence, transcribed products arising from ERVs may promote, or even be required for, the pluripotent state [24–33]. Finally, reports of L1 retrotransposition in somatic cells have fueled speculation that TE-derived mosaicism may lead to functional innovation during development [34–37].

Here, we review the restraint and activity of TEs in embryonic cells and later in development, as well as the unexpected promotion of pluripotent states by ERVs. We further appraise the convergent contributions to embryogenesis made by ERVs in distinct mammalian clades as evidence of an evolved strategy to avoid, or at least delay, host genome repression.

## ERV-driven transcription in the early embryo

### ERV regulation of protein-coding genes

Although there are spectacular examples of TE proteins underpinning functional innovation, such as in the placenta [38], regulatory sequences exapted from TEs arguably loom larger in our evolutionary history [15]. Indeed, up to 30 % of human and mouse transcription start sites (TSSs) are situated in TEs and display tissue-specific expression patterns [33, 39]. Embryonic human tissues express the greatest diversity of TE-associated TSSs observed to date [33], highlighting the potential of TEs to drive cell type and developmental stage-specific expression, particularly during early embryogenesis when the genome becomes demethylated [40]. In mouse, the LTR promoters of MuERV-L elements regulate a network of genes critical for totipotency and specific to the two-cell stage of embryonic development [41]. TE-derived regulatory sequences likewise contribute to the evolution of regulatory networks in pluripotent stem cells. For example, only about 5 % of Oct4 and Nanog transcription factor (TF) binding sites are shared in mouse and human embryonic stem cells (hESCs). TEs contribute a significant proportion (about 25 %) of the remaining, species-specific, binding sites [42]. Moreover, in vitro knockdown of specific ERVs via RNA interference can lead to a reduction in pluripotency markers [24, 26–28, 43–46]. Thus, TE sequences are broadly and strongly transcribed in the early embryo and can influence pluripotency by being exapted into, or at least adding robustness to, pluripotency networks. These findings underscore the universality and versatility of TEs in driving the evolution of regulatory networks.

### Independent ERV expression as a hallmark of the pluripotent state

ERV transcription independent of protein-coding genes has also been linked to pluripotency. Despite an apparent lack of retrotransposition activity, specific HERVs are actively transcribed in hESCs and are thought to influence pluripotency maintenance [24, 25, 27–32, 47]. The HERV families HERV-H and HERV-K (HML-2) in particular appear to be connected to early human embryonic development [25, 31]. While stochastic transcriptional derepression of various HERVs [47] as well as non-LTR retrotransposons [48] in pluripotent cells can probably be attributed to a general relaxation of TE silencing [40], specific classes of elements are consistently reactivated across hESC lines, indicating that their expression can serve as a marker for an undifferentiated state [28, 29], further raising the possibility that these elements have a functional link to pluripotency. Distinct HERV families also denote specific embryonic stages, suggesting HERV expression profiles may signify cell identity [25]. It is important to note, however, that, in many cases, only a small fraction of HERVs from a specific family are transcribed [25] and that their genomic context likely plays a pivotal role in their expression. The reasons for HERV families presenting distinct expression patterns during early embryogenesis are currently unclear. To speculate, such patterns could be a reflection of the optimal “ecological niche” of their ancestral exogenous counterparts and may mimic the parallel expression patterns of LTR-binding TFs.

Human oocytes and zygotes (to the cell–cell stage) contain the highest percentages of HERV transcripts observed during development; these are almost certainly deposited maternally prior to embryonic genome activation [25]. Abundant transcription emanating from MaLR and ERVK LTRs has also been documented for mouse oocytes [5, 49]. The provision of ERV transcripts by the maternal genome supports ERV functionality in the early embryo, as these RNAs already seem to be necessary before the embryonic genome is able to generate its own transcripts [31]. However, it is also possible that ERV transcripts do not have a specific function at this early stage but their maternal deposition is permitted because they do not harm the developing embryo. Nevertheless, stage-specific expression from ERV promoters, and of protein-coding genes, LTR-driven chimeric transcripts, and ERV transcripts proper, is a defining feature of early mammalian development.

## Regulation of HERV-K and HERV-H by pluripotency factors

**Fig. 4:**
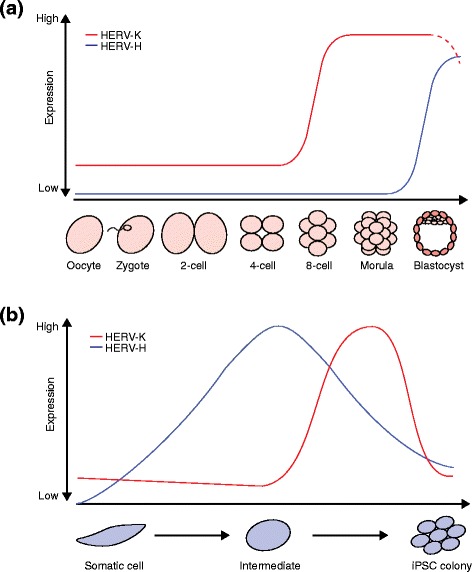
Human endogenous retrovirus (*HERV*) expression patterns in pluripotent cells. **a** HERV-K transcription in human embryogenesis is initiated during embryonic genome activation at the eight-cell stage and remains until the blastocyst stage. *Dashed lines* indicate proposed expression of HERV-K [31]. HERV-H can only be detected in epiblast cells of the late blastocyst [25]. **b** After induction of induced pluripotent stem cell (*iPSC*) reprogramming, HERV-K and HERV-H are derepressed with distinct dynamics. HERV-K transcription reaches its peak shortly before cells are fully reprogrammed. HERV-K expression subsequently decreases in reprogrammed cells and is silenced in iPSCs [32]. HERV-H is highly expressed earlier during reprogramming compared with HERV-K [24]. Note: the time points shown are approximate due to technical differences between studies

As well as gene regulation transacted by ERVs, many studies have revealed how ERVs are in turn regulated by pluripotency genes. For instance, the core pluripotency TFs Oct4 and Nanog (Box 1) bind specific HERV families (Fig. 3) [26, 42]. HERV-K is the most recently active HERV family and many HERV-K copies retain their protein-coding potential [50]. Notably, transcription from the youngest subclass of HERV-K is induced from its LTR, known as LTR5HS (for “human-specific”), at the eight-cell stage, during embryonic genome activation, and continues through to the blastocyst stage (Fig. 4a). LTR5HS contains an Oct4-binding motif that is not present in older LTRs such as LTR5a or LTR5b [31]. DNA hypomethylation and transactivation by Oct4 at LTR5HS synergistically stimulate HERV-K expression and lead to the presence of retroviral and viral-like particles in human preimplantation embryos [31]. HERV-K type 2 proviruses encode the protein Rec, which derives from alternative splicing of the *env* gene and is responsible for nuclear export and translation of viral RNAs [51]. Rec can be found in pluripotent cells and may influence expression of the interferon-induced viral restriction factor IFITM1 in epiblast cells [31, 52]. Consequently, Grow et al. [31] suggested that antiviral responses might be induced by HERV-K proteins, protecting the human embryo against new retroviral infections. Similarly, HERV-K type 1 proviruses encode the protein Np9, which is the product of a new alternative splicing event and coincides with a deletion in the *env* region [53, 54]. Interestingly, Rec and Np9 are not encoded in rodent ERVs, making them a distinguishing feature of primate ERVs and, moreover, hESCs specifically express Rec, Np9, and Gag [29]. It is tempting, therefore, to speculate, as per Grow et al. [31], that hESCs allow expression of these HERV-K proteins to fulfill a protective function via, for example, Rec-induced inhibition of viral infection. It is also possible that some HERV-K elements fortuitously escape silencing and manufacture viral proteins as innocuous byproducts of HERV-K transcription in hESCs (Fig. 3).

HERV-H is another primate-specific retrotransposon [55] with a potentially important role in the maintenance of hESC identity and pluripotency (Table 1). HERV-H transcripts are expressed in pluripotent cells at levels much higher than those seen in differentiated cells and, as a result, HERV-H expression is a proposed marker for pluripotency [28]. Interestingly, HERV-H is expressed in some induced pluripotent stem cell (iPSC) lines (Box 1) at higher levels than for other iPSC lines and embryonic stem cells (ESCs) [47]. Developmental HERV-H expression also appears to be cell type and stage specific in vivo (Fig. 4a). For instance, HERV-H and its flanking LTR element LTR7 can only be detected in epiblast cells [25], whereas other related LTR variants that flank HERV-H (LTR7B and LTR7Y) are detectable at the eight-cell stage and morula [25]. LTR7 incorporates Oct4, Nanog, Klf4, and Lbp9 TF binding sites, which together appear to mediate HERV-H transcriptional activation [28]. Once activated, individual LTR7 copies can generate noncoding RNAs [43] and form chimeric transcripts with protein-coding genes, in some cases supplying multiple promoters to the same gene (Fig. 3) [27, 28, 56]. LTR7 may also be bound by factors central to so-called naïve, or ground state, pluripotency where cells are predisposed to self-renew and lack differentiation markers, showing that ERVs may be involved in fine tuning stem cell phenotype [28, 57]. In sum, HERV-K and HERV-H are clearly activated by pluripotency TFs and their expressed products are, at the very least, markers of pluripotency.

**Table 1:** Summary of HERV-H findings to date in human stem cells

Findings	Reference(s)
Binding of pluripotency TFs in ESCs and iPSCs within or near LTRs or specific LTR-driven lncRNAs	[24, 28, 42–45, 47]
Induction of HERV-H, or LTR7-driven lncRNA/chimeric RNA expression, in ESCs, declining upon differentiation	[26–28, 43–45, 47, 60, 161]
Active chromatin marks on specific LTRs in ESCs or iPSCs	[28, 32, 43, 44, 47, 162]
DNA hypomethylation at specific LTRs in ESCs	[28, 161]
LTR enhancer activity in ESCs	[27]
Changes in HERV-H associated with expression changes of genes near LTRs	[27, 28, 32, 42]
Induction of HERV-H in iPSCs, declining upon differentiation	[24, 27, 28, 32, 46]
Differentiation-defective iPSC clones retain high levels of HERV-H RNA	[46]
Knockdown of general HERV-H expression inhibits iPSC formation and causes ESC differentiation	[24, 27, 28]
Knockdown of specific LTR-driven RNAs inhibits iPSC formation or causes ESC differentiation	[24, 28, 44, 45]
LTR-driven lncRNA acts as miRNA sponge to positively regulate pluripotency TFs	[45]
HERV-H RNA associates with coactivators and LTR loci in ESCs	[27]
HERV-H expression marks for naïve-like stem cells	[28]
HERV-H LTR subtypes expressed sequentially in early development	[25, 31]

*ESC* embryonic stem cell, *HERV* human endogenous retrovirus, *iPSC* induced pluripotent stem cell, *lncRNA* long noncoding RNA, *LTR* long terminal repeat, *miRNA* microRNA, *TF* transcription factor

## HERV-derived long noncoding RNAs regulate pluripotency networks

Long noncoding RNAs (lncRNAs) are RNA transcripts greater than 200 nucleotides long that possess no, or very little, protein-coding potential [58–60]. Most lncRNAs are transcribed antisense to protein-coding genes or are intergenic [58, 59]. More than two-thirds of lncRNAs incorporate TE sequences (Fig. 3) and, in cases such as *Xist*, a prototypical lncRNA involved in X chromosome inactivation, TEs are a core component of lncRNA biogenesis [60, 61]. Other than *Xist*, and a few additional examples, lncRNAs have proven difficult to evaluate functionally because, as well as containing TEs, lncRNAs are often expressed at very low levels [30]. However, one of the best established lncRNA functions is to regulate pluripotency, particularly by mediating changes to chromatin [62, 63]. Interestingly, Au et al. [64] reported more than 2000 additional long intergenic noncoding RNA (lincRNA) isoforms, of which 146 were expressed in hESCs. These human pluripotency-associated transcripts (HPATs) typically incorporated ERVs, especially HERV-H [30], and in that regard were similar to many other hESC-specific lncRNAs [27, 43, 44, 47]. HPATs appear to contribute to formation of the blastocyst ICM, suggesting an essential role for HERV-derived lncRNAs in human embryogenesis [30].

One particularly interesting lincRNA, HPAT5, is hypothesized to be involved in post-transcriptional gene regulation: HPAT5 binds AGO2, a core protein catalyzing microRNA (miRNA) processing [65], and the let-7 miRNA family, which modulates hESC pluripotency [66]. Durruthy-Durruthy et al. [30] have suggested that HPAT5 controls the balance between pluripotency and differentiation by negatively regulating let-7 expression. However, HPAT5 is promoted by the so-called HUERS-P1 ERV, a low copy number TE that has not been investigated very deeply in this context. Interestingly, the HPAT5 promoter is located in the internal Gag sequence of the HUERS-P1 ERV, rather than in an LTR. Therefore, this promoter likely developed by genetic drift or selection, rather than by harnessing the “ready to use” regulatory motifs found within an LTR. In addition, the let-7 binding site within HPAT5 occurs within an imbedded *Alu* element. HPAT5 is thus an unusual, and yet fascinating, example of retrotransposon-driven regulatory innovation.

More broadly, HERV-driven transcripts contributing to pluripotency networks unique to humans or primates are of particular interest. *lincRNA-RoR*, with its TSS located in a HERV-H element, represents an excellent example of a primate-specific TE found to modulate pluripotency [43]. Notably, *lincRNA-RoR* is expressed more highly in iPSCs than in ESCs and can promote iPSC reprogramming [44], perhaps by serving as an miRNA sponge protecting Sox2 and Nanog from miRNA-mediated degradation [45]. In another example, the gene *ESRG*, which uses a domesticated HERV-H promoter, plays a role unique to human pluripotency [28]. Unusually, *ESRG* encodes an intact open reading frame (ORF) in humans, but possibly not in other primates, and is expressed exclusively in the human ICM and cultured pluripotent cells [67]. *ESRG* knockdown compromises stem cell self-renewal and promotes differentiation, while *ESRG* overexpression aids reprogramming [28]. These case studies demonstrate recurrent incorporation of annotated HERV-derived transcripts into pluripotency networks.

To discover new lncRNAs regulating pluripotency, Fort et al. [26] surveyed in depth the noncoding transcriptomes of mouse and human stem cells. The resulting pluripotency lncRNA catalog included numerous previously unreported antisense, intergenic, and intronic transcripts that initiate in ERVs. Consistent with an earlier report [33], Fort et al. found an exceptional variety of stem cell-specific TSSs that are not directly associated with protein-coding genes. These TSSs often overlap with TEs, especially with ERVK and MaLR LTR subfamilies in mice and ERV1 in humans, and frequently flank enhancer elements. In addition to bidirectional transcription denoting enhancer activity [68, 69], TE-derived enhancer sequences are enriched for bound Nanog, Sox2, Oct4, and the enhancer-related protein p300 [26]. As such, regulation of TE-derived enhancers and lncRNAs by pluripotency TFs can result in the formation of positive-feedback loops, potentially bolstering pluripotency networks [25, 26, 62]. Thus, in agreement with other studies, Fort et al. demonstrated that specific ERVs are major contributors to the stem cell transcriptome and found a plethora of novel stem cell-associated ERV-derived transcripts that await functional characterization, in line with expectation that some of these lncRNAs will be involved in the establishment and maintenance of pluripotency [70].

## ERV expression dynamics during somatic cell reprogramming

Domesticated TEs clearly play important functional roles in stem cell biology. However, TE repression can shift as cells transition through pluripotent states, as encountered during reprogramming. As a result, opportunistic TEs may mobilize, cause insertional mutagenesis and, potentially, compromise the integrity of reprogrammed cells [32, 48, 71]. TE activity in stem cells therefore carries risk as well as benefits for the host genome, along with major incentives for TEs, given potential for early embryonic retrotransposition events to be germline transmitted. It follows that, although reprogramming can broadly reactivate TEs, particularly those controlled by TFs expressed dynamically during reprogramming [16, 42], silencing is selectively re-established in the resulting pluripotent cells, potentially ameliorating risk to the host genome. For instance, although HERV-H and HERV-K are both transcriptionally active during reprogramming, HERV-H is expressed in cultured iPSCs, whereas the more recently mobile HERV-K family is silenced [28] (Fig. 4b). This contrast is also found for mouse iPSCs, where Mus type-D related retrovirus (MusD) expression contrasts with intracisternal A-type particle (IAP) silencing [32]. Importantly, more experiments are required to confirm the generality of these observations, as technical considerations in iPSC generation (e.g., reprogramming and culture conditions) can lead to differences in TE expression between iPSC lines [71].

TE repression is dynamic during reprogramming. In a high-resolution analysis of mouse and human iPSC lines, Friedli et al. [32] found that most ERVs peaked in expression shortly before reprogramming was complete and were then repressed in pluripotent cells. Broad TE expression during somatic cell reprogramming may be in itself important for the induction of the pluripotent state. Ohnuki et al. [24] reported, for example, that LTR7 elements (associated with HERV-H) are hyperactivated by Oct4, Sox2, and Klf4 during reprogramming. In the resultant iPSCs, however, LTR7 activity decreased to levels seen in hESCs and, notably, ectopic LTR7 hyperactivity in iPSCs resulted in a differentiation-defective phenotype [24]. Similarly, cumulative HPAT expression rises markedly during reprogramming and is diminished in iPSCs and, as for HPAT5, may influence reprogramming efficiency [30]. Taken together, these data indicate that TE hyperactivity is potentially deleterious to the host genome due to an elevated risk of retrotransposition but may also be a requirement of induced reprogramming.

## ERV silencing in pluripotent states

The machineries responsible for ERV regulation in ESCs are evidence of the complex relationships that can form between TEs and their host genome. Broadly speaking, to reduce the probability of retrotransposon-derived mutagenesis, mammalian genomes target ERVs with DNA methylation, heterochromatin-forming factors, transcriptional repressor complexes, proviral silencing factors, and post-transcriptional arrest or degradation of viral RNAs (Table 2) [19, 20, 72]. Prominently, histone modifications silence ERVs in ESCs [73–75] by making chromatin inaccessible to polymerases and transcription factors [76], although this silencing in itself carries potential for deleterious side effects when nearby genes are also inadvertently repressed [77]. Moreover, some ERVs are marked by H3K9me3 and H4K20me3 for repression in ESCs but not in differentiated cells [6], suggesting that this pathway is used for de novo establishment of heterochromatin around ERV sequences [75, 78] or, alternatively, is used to maintain repression already established in oocytes [79, 80].

**Table 2:** Selected factors silencing ERVs in embryonic stem cells

Factor	Relevant function in ESCs	Interacting proteins	Reference(s)
Zfp809	KRAB zinc finger protein, recognizes PBS Pro and recruits Trim28	Trim28	[85]
YY1	Yin-Yang 1 (YY1) is a zinc finger protein, initially binds to mouse ERV LTRs (U3 region) and helps assemble Trim28/Eset silencing complex	Trim28	[92]
SHIN	Short heterochromatin inducing sequence initiates heterochromatin and transcriptional repression	KRABZfps (likely)	[93]
Trim28 (Kap1, Tif1-β)	Transcriptional co-repressor, acts as a bridge between KRAB-Zfps and other transcriptional repressors, mediates H3K9me3 recruitment	KRABZfps, Eset, NuRD deacetylase complex, HP1	[74, 86]
Eset (Setdb1, Kmt1e)	Histone methyltransferase, trimethylates H3K9 and H4K20, crucial for HP1 binding	Trim28, HP1	[73]
Sumo2	Sumoylation factor, post-translational sumoylation of Trim28 enhances recruitment of Trim28 to proviral DNA	Trim28	[72]
HP1	Heterochromatin protein 1, binding to Trim28 might be important for repression of transcription	Trim28, Eset, Atrx	[86, 89]
Chaf1a	Histone chaperone, deposits histone H3/H4 which marks proviral DNA for silencing, might execute different silencing mechanisms on different classes of ERVs	Eset, Kdm1a, Hdac1/2, Asf1a/b	[72]
Asf1a/b	Histone chaperones, components of the Chaf1a interactome	Chaf1a	[72]
Atrx	ATP-dependent helicase, establishes and maintains heterochromatin	Eset, Trim28 (both likely)	[93]
Daxx	H3.3-specific chaperone, may facilitate H3.3-deposition on ERV sequences, possible role in SHIN-silencing	Atrx	[93, 95]

*ERV* endogenous retrovirus, *ESC* embryonic stem cell, *KRAB* Krüppel-associated box, *LTR* long terminal repeat, *SHIN* short heterochromatin inducing sequence, *Zfp* zinc finger protein

Even ERVs in accessible chromatin can be decisively silenced by DNA methylation. In mice, de novo DNA methylation is regulated by the canonical Zfp/Trim28/Eset machinery [75]. Krüppel-associated box (KRAB) zinc finger proteins (Zfps) play a major role in the initiation of ERV silencing [81, 82]. Indeed, the number of ERVs and Zfp genes in vertebrates are correlated, suggesting coevolution [83]. As an example of the complexity of Zfp-mediated retrovirus silencing, Zfp809 knockout induces the in vivo expression of Moloney murine leukemia virus (MMLV)-like 30 (VL30) provirus [84]. Zfp809 also binds to MMLV and initiates silencing by recruiting Trim28 (also known as Kap1) [74, 85, 86]. Trim28 activity is enhanced by post-translational sumoylation by Sumo2 [72, 87] and binds HP1, which is thought to contribute to the ability of Trim28 to repress transcription in the context of MMLV silencing [86, 88, 89]. Another Zfp, YY1, also binds to MMLV [90, 91] and, together with Zfp809, is thought to recruit Trim28 to ensure a stably DNA-bound silencing complex [92]. In another example, KRAB Zfps have been shown to trigger heterochromatin formation in IAP retrotransposons by binding to a short heterochromatin inducing (SHIN) sequence, dependent on Eset and Trim28 [93], enacting H3K9 and H4K20 trimethylation [73]. Chaf1a facilitates deposition of these H3 and H4 variants and also interacts with Eset [72]. Eset-mediated ERV silencing is also important in mouse primordial germ cells before the onset of de novo DNA methylation [80]. Hence, ERV silencing is enacted by a multilayered and interleaved system that ensures robust and specific repression of ERV families, subsets, and individual loci.

**Fig. 5:**
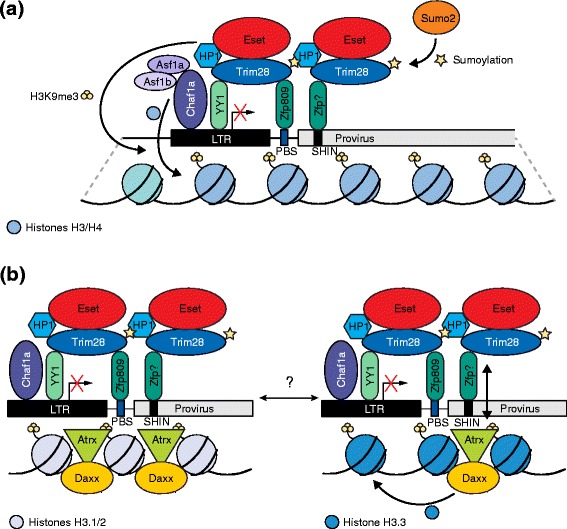
Proposed models of de novo endogenous retrovirus (*ERV*) silencing in embryonic stem cells. **a** To initiate silencing, the Krüppel-associated box (KRAB) zinc finger protein (*Zfp*) Zfp809 interacts with the proline primer binding site (PBS Pro) of some ERV families (e.g., Moloney murine leukemia virus) [85] whereas other KRAB-Zfps bind to a short heterochromatin-inducing (*SHIN*) sequence found in intracisternal A-type particle retrotransposons and other ERV families [93]. Subsequently, Trim28 is recruited by the Zfps [74, 86], assisted by binding of YY1 to the long terminal repeat (*LTR*) and Trim28 [92]. Interaction with HP1 and sumolyation by Sumo2 are thought to contribute to transcriptional repression mediated by Trim28 [72, 86, 89]. Eset also interacts with Trim28 and enables trimethylation of H3K9 and H4K20 [73]. The histone chaperone Chaf1a, aided by Asf1a/b, marks proviral DNA for silencing by depositing histones H3 and H4 and interacts with Eset [72]. **b** Conflicting models of ERV silencing by H3.3 deposition. The Atrx–Daxx complex is suggested to play an important role in SHIN-mediated silencing, which is H3.3-independent. Here, Atrx is thought to promote ERV heterochromatin inaccessibility (*left*) [93]. However, Atrx–Daxx is also proposed to deposit H3.3 and to interact with Trim28, followed by H3.3 being marked with H3K9me3 by Eset (*right*) [95]

It follows that models explaining ERV silencing are typically complex, which, at times, can lead to differing conclusions. For instance, the SNF2-type chromatin remodeler Atrx is another crucial component for IAP silencing that renders Eset-dependent heterochromatin less accessible [93] and is likely to be recruited to IAPs by Trim28 and Eset [93] (Fig. 5a). Interestingly, Atrx has been reported to interact with the H3.3-specific chaperone Daxx to facilitate H3.3 deposition at telomeric heterochromatin [94]. Yet, it is not clear if H3.3 is required for ERV silencing, despite detection of H3.3 across ERV flanking regions and solo LTRs [95]. In general, Sadic et al. [93] and Elsässer et al. [95] reached opposing conclusions with regards to H3.3 enrichment around ERV sequences (Fig. 5b). One possible explanation here is that Elsässer et al. used chromatin immunoprecipitation sequencing (ChIP-seq) to detect H3.3-enriched regions across the entire mouse genome and found a correlation between H3.3, H3K9me3, and ERV coordinates. Sadic et al., on the other hand, used an engineered reporter assay to measure ERV silencing which, in H3.3 knockout cells, remained intact. Further study is therefore required to resolve the place of H3.3 in models of ERV silencing. Overall, these and other examples of TE repression in pluripotent cells, such as the silencing of nascent L1 and MMLV insertions in embryonic carcinoma derived cell lines [96, 97], reflect the extraordinary efforts made by the host genome to orchestrate silencing of currently and recently retrotransposition-competent TEs during embryonic development.

## Endogenous L1 mobilization in mammalian somatic cells

The early embryo is a viable niche for the generation of potentially heritable retrotransposon insertions. In particular, L1 mobilization in human and rodent embryos may drive somatic and germline mosaicism [98–101] and, indeed, deleterious human L1 insertions transmitted from mosaic parents to offspring have resulted in sporadic genetic disease [101]. In vitro experiments have likewise provided support for L1 mobilization occurring in pluripotent cells [99–101] and, potentially, the presence of the L1 retrotransposition machinery being required for preimplantation mouse embryo development [102]. Human iPSCs and ESCs allow low-level mobilization of an engineered L1 reporter [22, 48, 99]. Consistently, endogenous L1 promoter hypomethylation and transcriptional activation have been observed in iPSCs [32, 48, 71], as has induction of a primate-specific L1 antisense peptide (ORF0p) that appears to increase L1 mobility in stem cells [56] (Box 2). Endogenous de novo L1 retrotransposition and mobilization of nonautonomous *Alu* and SINE–VNTR–*Alu* (SVA) elements have also been reported by Klawitter et al. [71] in several iPSC lines, as well as an *Alu* insertion in a cultured hESC line. L1 may, therefore, *trans* mobilize *Alu* and other SINEs during development, an important finding due to the high potential of SINEs to impact gene regulation [12, 71, 103, 104]. Klawitter et al. estimated that approximately one de novo L1 insertion occurred per cell in human iPSCs. Strikingly, more than half of the detected de novo L1 insertions were full length and thus potentially able to mobilize further. Klawitter et al. also observed extraordinary induction of L1 mRNA and protein expression after reprogramming. To speculate, numerous L1 ribonucleoprotein particles (RNPs; Box 2) could form as a result and be carried through iPSC culture and differentiation. This could enable L1-mediated insertional mutagenesis in cells descending from those where L1 expression originally occurred, as others have considered for L1 RNPs arising in gametes and carrying over into the zygote [100].

**Fig. 6:**
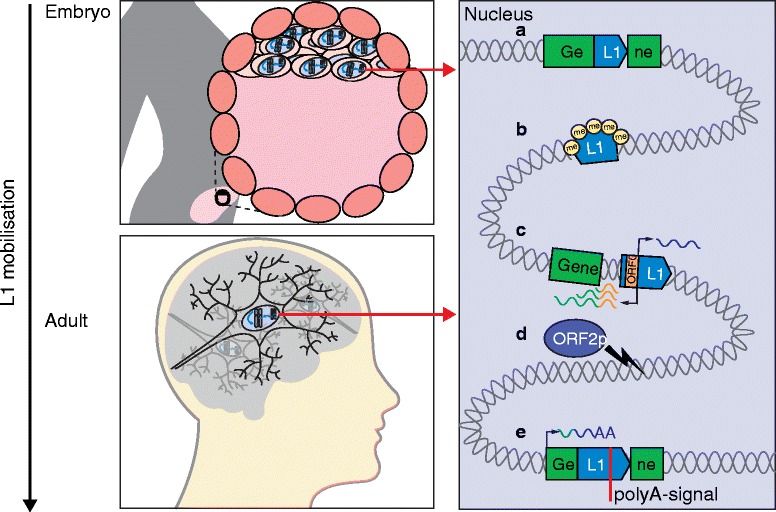
Long interspersed element-1 (*L1*) contributes to somatic mosaicism. L1 mobilizes in the brain and early embryo (*left*) and may, for example: **a** insert into protein-coding exons; **b** influence neighboring genes by the spreading of repressive histone modifications, such as methylation (*me*); **c** initiate sense or antisense transcription of neighboring genes, thereby creating new transcripts, including open reading frame 0 (*ORF0*) fusion transcripts, using host gene provided splice acceptor sites, which are translated to fusion proteins; **d** generate DNA double-strand breaks via the endonuclease activity of L1 ORF2p; and **e** lead to premature termination of host gene transcripts by providing alternative poly(A) signals

Although both L1 and ERV retrotransposons are active in the mouse germline [105, 106], their capacity to mobilize during embryogenesis is less clear than for human L1. Quinlan et al., for instance, concluded de novo retrotransposition in mouse iPSCs did not occur, or was very rare [107], in contrast to results for human iPSCs [22, 48, 71]. However, an earlier study found that engineered L1 reporter genes mobilize efficiently in mouse embryos [100]. Interestingly, the vast majority of engineered L1 insertions in these animals were not heritable, perhaps indicating retrotransposition later in embryogenesis [100]. Targeted and whole-genome sequencing applied to mouse pedigrees has, conversely, revealed that endogenous L1 mobilization in early embryogenesis is relatively common and often leads to heritable L1 insertions (SRR and GJF, unpublished data). Polymorphic ERV and nonautonomous SINE insertions are also found in different mouse strains [105, 106]. Although the developmental timing of these events is as yet unresolved, we reason that they can occur in spatiotemporal contexts supporting L1 retrotransposition. It follows that both human and mouse L1s, and probably mouse ERVs, can mobilize in embryonic and pluripotent cells (Fig. 6), as well as gametes. The resultant mosaicism can be deleterious to the host organism or their offspring [101], again reinforcing the need for TE restraint during early development.

Somatic L1 retrotransposition can also occur later in development. Over the past decade, it has become accepted that the mammalian brain, particularly cells of the neuronal lineage, accommodate mobilization of engineered and endogenous L1 elements [34–37, 108]. Although the frequency of somatic L1 insertions during neurogenesis is disputed [35, 36, 108, 109], this is largely due to differences in the advanced techniques required to discriminate genuine de novo L1 insertions and molecular artifacts arising during whole-genome amplification of individual human neurons. This discrimination can, broadly, be achieved quantitatively, by assuming true-positives will accrue more DNA sequencing reads than artifacts [108], or qualitatively, by analyzing the junction DNA sequences between putative L1 insertions and the flanking genome and excluding examples inconsistent with target-site primed reverse transcription [35]. Despite this debate, there is agreement that L1 mobilization occurs in the brain and can, for the most part, be traced to neuronal precursor cells [35, 36, 109]. Remarkably, neuronal L1 insertions are distributed unevenly genome-wide and are enriched in neurobiological genes and transcribed neuronal enhancers [34, 35]. Somatic L1 insertions oriented in sense to host genes, as the configuration most likely to disrupt transcription [110, 111], are heavily depleted versus random expectation, providing possible evidence of selection against these events during neurogenesis [35]. Concordantly, somatic L1 insertions in neurobiological genes carry an elevated chance of yielding a molecular phenotype in the brain, especially provided the numerous routes by which L1 insertions can profoundly modify gene structure and expression (Fig. 6) [12, 33, 77, 110, 112–118].

Neuronal L1 insertions impart no obvious evolutionary benefit as they cannot be transmitted to subsequent generations. Thus, it is tempting to speculate that L1 activity is derepressed during neuronal commitment to serve a biological purpose for the host organism, analogous to the potential exaptation of ERV transcription for pluripotency maintenance and following the example of the vertebrate adaptive immune system, where domesticated TEs mediate V(D)J recombination and functional diversification through genomic mosaicism [119]. Similarly, although individual somatic L1 insertions in neurons are not inherited, it is plausible that the cellular mechanisms and factors enabling their production may undergo evolutionary selection [109]. While L1-mediated somatic mosaicism in neurons may eventually be shown to have functional or behavioral consequences [109, 118], numerous additional experiments are required to assess this hypothesis. Whether perturbation of L1 regulation and retrotransposition in the brain is connected to neurological disease is not yet clear [35, 120–122]. The available evidence does, however, show conclusively that TE mobilization occurs during embryogenesis and, in a more restricted fashion, later in life.

## Conclusions

The mammalian genome clearly strives to limit TE activity in pluripotent cells. The silencing mechanisms involved are collectively complex and broadly potent and yet are also capable of great specificity and dynamism in targeting individual TE copies [17]. In this regard, ERVs present two contrasting facets: firstly, the control mechanisms that have evolved to restrict ERV activity and, secondly, the domestication of ERV sequences into pluripotency maintenance. Specific ERV families, such as HERV-H and HERV-K, can provide binding sites for pluripotency TFs, produce stem cell-specific protein-coding and noncoding transcripts, and harbor new enhancers. Over time, these contributions have led to the integration of ERVs into gene networks governing embryogenesis and, surprisingly, independent ERV hyperactivity appears to be a harbinger of pluripotent states. Conversely, notwithstanding a need for more experimental data for murine ERVs, L1 appears to be the most successful TE to mobilize in mammalian somatic cells and, at the same time, is arguably less likely to impact their phenotype than ERVs (Fig. 2). During human iPSC reprogramming, for example, L1 and ERVs can both be broadly derepressed, but with divergent repercussions for the host genome and providing different opportunities to each TE family.

Why are TEs active, and apparently essential, in the embryo? The relationship between TEs and the host genome is often referred to as an evolutionary arms race [123, 124]. A review specifically addressing the role of TEs in pluripotency [14] refined this concept to more of a genetic conflict of interest between ERVs and the host genome, where exposure to retrotransposition was a necessary risk of the pluripotent state. The authors, as others have done [28], also considered the possibility that ERVs were active in stem cells by serendipity. Despite their merits, each of these alternatives is contradicted by several considerations. Firstly, L1 mobilization appears to be far more common in the embryo than ERV mobilization, despite ERV domestication being overtly more useful to the host given the many ways ERVs can reinforce pluripotency (Fig. 3). The benefits of unleashing L1 and ERV activity do not seem then, in either case, to be commensurate with the implied risk of doing so. Secondly, ERVs are intrinsic to the pluripotent state but are now almost, if not fully, immobile in humans. Thirdly, different ERV families are centrally involved in human and mouse pluripotency; convergent evolution driven by the common environmental demands of embryonic development, which are conserved among mammals, is an improbable outcome of chance. Here, time and scale are critical considerations: the vast majority of new ERV insertions will be immediately silenced but, as the retrotranspositional potential of an ERV family is eliminated over time via mutations, pressure to silence the associated LTRs may also diminish, allowing them to regain their regulatory activity. Hence, with sufficient time, distinct ERV families in different species can ultimately come to occupy similar niches, in pluripotency and elsewhere. TEs pervade mammalian genomes and, as such, even the low probability of a de novo ERV insertion immediately escaping silencing presents a reasonable overall chance of such events becoming important to genome-wide regulation. This remains true even if the ERV family is eventually immobilized.

Although not rejecting models based on serendipity or conflict, we highlight that ERVs and other successful TE families commonly arise as low copy number families and then rapidly expand over generations. This scenario could lead to TEs acquiring traits of early pioneers in a potentially hostile genomic landscape. Two not necessarily exclusive strategies can aid TE survival in this environment. One is stealth. For example, adaptation of the L1 5′ promoter (Box 2) enables evasion of host genome surveillance, leading to continuing L1 retrotransposition during development. That most new L1 copies are 5′ truncated, and lack the canonical promoter, also reduces their visibility to surveillance. Although this self-limits the capacity of new L1 insertions to retrotranspose, it also reduces pressure on the host genome to clamp down on L1 activity. The other strategy is gaining acceptance by being useful. ERV promoters are repeatedly found in pluripotency regulatory networks and may, therefore, be intrinsic to the pluripotent state. In this setting, efforts by the host genome to limit ERV activity could be detrimental to pluripotency. As such, ERVs may be able to propagate for longer than would be possible should the host engage in resolute inhibition. Importantly, these strategies are predicated on embryonic retrotransposition having potential for germline transmission, i.e., carrying risk for host genome integrity, as many studies have now found. Even after ERV families are no longer capable of mobilization, their inherent capacity for regulation, especially by solo LTRs, is retained and provides a long-term evolutionary incentive for the host genome to maintain at least one active TE family, as almost all mammals do. As such, rather than an arms race, conflict, or even symbiotic relationship, we would propose that pioneer ERVs adopt peaceful survival strategies and that intricate mechanisms for TE repression have evolved to allow the host genome to harness those strategies over time, allowing some ERV families to expand and, as witnessed in the embryo, securely embed themselves by becoming indispensable. In advocating this model, we emphasize that indispensability of ERV-mediated regulatory effects in natural pluripotency and embryogenesis in vivo is still an open question. While difficult to pursue in humans, genetic knockout or deletion of individual mouse ERVs or ERV families implicated in pluripotency is possible [125] and, indeed, ultimately necessary to demonstrate their functional importance to the embryo.

## Box 1. Regulatory networks controlling pluripotency

Programmed shifts in transcriptional and epigenetic states during embryogenesis have been studied primarily using in vitro systems. Embryonic stem cells (ESCs) are pluripotent cells derived from the blastocyst inner cell mass. Cultured ESCs are intensively used to study pluripotency, particularly in humans. Over the past decade, a core regulatory circuit incorporating the transcription factors Oct4 (also known as Pou5f1), Sox2, and Nanog [126–128] has been revealed to regulate ESC pluripotency [129]. This circuit activates pluripotency-associated factors and represses lineage-specific genes [130]. Pluripotent cells can also be derived in vitro via somatic cell reprogramming. Induced pluripotent stem cells (iPSCs) were initially produced by forced expression of Oct4, Sox2, Klf4, and c-*Myc* using retroviral vectors [131, 132]. Numerous methods have since been developed to improve reprogramming efficiency and iPSC safety [133]. As for ESCs, iPSCs provide a powerful system to understand the pluripotent state and can differentiate to all cell types of the body [131, 132]

## Box 2. L1 retrotransposons

The non-long terminal repeat retrotransposon long interspersed element-1 (L1) is the only autonomous, mobile human transposable element [10, 12, 116, 134]. L1 occupies approximately 17 % of the human genome [7]. L1 also mobilizes *Alu* and SINE–VNTR–*Alu* (SVA) elements in *trans* [135, 136]. Mice, by contrast, have three L1 subfamilies (T_F_, G_F_, and A) that are autonomous, as well as nonautonomous short interspersed elements (SINEs) retrotransposed by L1 [10]. L1 accounts for 19 % of the mouse genome [8]. A full-length human L1 is approximately 6 kb long and initiates mRNA transcription from a 5′ sense promoter active in gametes, stem cells, and various somatic tissues [33, 36, 48, 71, 137–139]. The bicistronic L1 mRNA encodes two proteins, ORF1p and ORF2p, which are flanked by 5′ and 3′ untranslated regions (Fig. 1a). An L1 antisense peptide (ORF0p) [56] can also be expressed by an adjacent L1 antisense promoter [115]. This antisense promoter is expressed in many spatiotemporal contexts, including in stem cells, and can provide alternative promoters to protein-coding genes [33, 56, 115, 140]. L1 ORF2p presents endonuclease [141] and reverse transcriptase [142] activities and, during retrotransposition, L1 ORF1p, ORF2p, and the canonical L1 mRNA associate in *cis* to form a cytoplasmic ribonucleoprotein particle (RNP) [143]. The RNP can then enter the nucleus, where the ORF2p endonuclease cleaves genomic DNA, and the ORF2p reverse transcriptase synthesizes a new L1 copy at the cleavage site using the L1 mRNA as a template. This process is called target-site primed reverse transcription (TPRT) [144] (Fig. 1c).

The L1 5′ promoter is the major focus of host genome efforts to prevent L1 mobility, through DNA methylation and transcription factor repression and other pathways [145, 146]. Thus, it appears that L1 in the main persists as a mobile element by avoiding detection of its 5′ promoter by host genome surveillance pathways and, where this fails, by harnessing new promoter structures [13]. This could explain the exceptional L1 5′ promoter diversity observed even among closely related primates [23]. It should also be noted that the vast majority of L1 copies in the genome are 5′ truncated and lack the 5′ promoter [13], meaning that the host factors guarding against full-length L1 transcription are not necessarily able to recognize truncated L1s.

## Box 3. Endogenous retroviruses

Endogenous retroviruses (ERVs) are derived from exogenous retroviruses that, at some point, infected an individual organism’s germ cells, integrated into their genome, and were subsequently inherited by their offspring. ERVs are divided into class I, class II, and class III elements, based on the exogenous virus class that they are most similar to [11]. Full-length ERVs are 5–10 kb in length, encode proteins important for mobilization, and are flanked by two identical long terminal repeats (LTRs; 300–1000 bp) that regulate ERV transcription. Loss of the *env* gene, found in exogenous retroviruses, is a common feature of ERVs as they adopt an intracellular life cycle as a retrotransposon [11, 147, 148]. ERV retrotransposition is initiated by the transcription of the 5′ LTR and terminates in the 3′ LTR, generating a terminally redundant mRNA that is translated into Gag and Gag–Pro–Pol fusion proteins. Gag proteins encapsulate the mRNA and fusion protein. Pro has protease activity whereas Pol possesses reverse transcriptase, ribonuclease, and integrase domains that generate independent proteins by proteolytic maturation. Together they produce a double-stranded cDNA copy of the ERV and flanking LTRs. This cDNA is then integrated into the genome by the ERV integrase [149] (Fig. 1b).

Human endogenous retroviruses (HERVs) comprise about 8 % of the human genome [7]. All HERVs are considered to be now retrotransposition incompetent [150, 151]. The HERV-K (HML-2) family is exceptional, with several members arising after the divergence of humans and chimpanzees (approximately 6 million years ago) and a handful of polymorphic HERV-K insertions found in human populations [152–155]. Although a mobile HERV-K element has yet to be identified in humans, it is possible that rare, as yet undiscovered polymorphic elements could retain retrotransposition competence [152]. In contrast to humans, ERVs account for approximately 10 % of the mouse genome [8]. Several mouse ERV families are still autonomously active, including intracisternal A-type particle elements [106], Moloney murine leukemia virus [156], and Mus type-D related retrovirus (MusD) [147] elements, as well as the MusD-dependent early retrotransposon family [157]. Together, new mouse ERV insertions are responsible for about 10 % of documented germline mutations in inbred strains [106]. Clade-specific ERVs also occur in other mammals, although genomic ERV content varies significantly between species [11]. Numerous instances of mammalian ERVs contributing regulatory sequences to genes, including examples of convergent evolution [158], are found in pluripotent cells and elsewhere [15, 159, 160].

## Abbreviations

ERV: endogenous retrovirus
ESC: embryonic stem cell
HERV: human endogenous retrovirus
hESC: human embryonic stem cell
HPAT: human pluripotency-associated transcript
IAP: intracisternal A-type particle
ICM: inner cell mass
iPSC: induced pluripotent stem cell
KRAB: Krüppel-associated box
L1: long interspersed element-1
lincRNA: long intergenic noncoding RNA
LINE: long interspersed element
lncRNA: long noncoding RNA
LTR: long terminal repeat
miRNA: microRNA
MMLV: Moloney murine leukemia virus
ORF: open reading frame
RNP: ribonucleoprotein particle
SINE: short interspersed element
TE: transposable element
TF: transcription factor
TSS: transcription start site
Zfp: zinc finger protein
